# Individual-fMRI-approaches reveal cerebellum and visual communities to be functionally connected in obsessive compulsive disorder

**DOI:** 10.1038/s41598-020-80346-6

**Published:** 2021-01-14

**Authors:** Rajan Kashyap, Goi Khia Eng, Sagarika Bhattacharjee, Bhanu Gupta, Roger Ho, Cyrus S. H. Ho, Melvyn Zhang, Rathi Mahendran, Kang Sim, S. H. Annabel Chen

**Affiliations:** 1 Centre for Research and Development in Learning (CRADLE), Nanyang Technological University, CRADLE, 61 Nanyang Drive, ABN-01b-10, Singapore, 637335 Singapore; 2 Department of Psychiatry, New York University School of Medicine, New York, USA; 3 Division of Clinical Research, Nathan S. Kline Institute for Psychiatric Research, Orangeburg, USA; 4 School of Social Sciences (SSS), Nanyang Technological University, 48 Nanyang Ave, SHHK-04-19, Singapore, 639818 Singapore; 5 Community Psychiatry, Institute of Mental Health, Singapore, Singapore; 6 Psychological Medicine, National University Health Systems, Singapore, Singapore; 7 Academic Development Department, Duke-NUS Medical School, Singapore, Singapore; 8 West Region, Institute of Mental Health, Singapore, Singapore; 9 Lee Kong Chian School of Medicine (LKC Medicine), Nanyang Technological University, Singapore, Singapore; 10 Office of Educational Research, National Institute of Education, Nanyang Technological University, Singapore, Singapore

**Keywords:** Neuroscience, Neurology

## Abstract

There is significant interest in understanding the pathophysiology of Obsessive–Compulsive Disorder (OCD) using resting-state fMRI (rsfMRI). Previous studies acknowledge abnormalities within and beyond the fronto-striato-limbic circuit in OCD that require further clarifications. However, limited information could be inferred from the conventional way of investigating the functional connectivity differences between OCD and healthy controls. Here, we identified altered brain organization in patients with OCD by applying individual-based approaches to maximize the identification of underlying network-based features specific to the OCD group. rsfMRI of 20 patients with OCD and 22 controls were preprocessed, and individual-fMRI-subspace was derived for each subject within each group. We evaluated group differences in functional connectivity using individual-fMRI-subspace and established its advantage over conventional-fMRI methodology. We applied prediction-based approaches to highlight the group differences by evaluating the differences in functional connections that predicted the clinical scores (namely, the Obsessive–Compulsive Inventory-Revised (OCI-R) and Hamilton Anxiety Rating Scale). Then, we explored the brain network organization of both groups by estimating the subject-specific communities within each group. Lastly, we evaluated associations between the inter-individual variation of nodes in the communities to clinical measures using linear regression. Functional connectivity analysis using individual-fMRI-subspace detected 83 connections that were different between OCD and control groups, compared to none found using conventional-fMRI methodology. Connectome-based prediction analysis did not show significant overlap between the two groups in the functional connections that predicted the clinical scores. This suggests that the functional architecture in patients with OCD may be different compared to controls. Seven communities were found in both groups. Interestingly, within the OCD group but not controls, we observed functional connectivity between cerebellar and visual regions, and lack of connectivity between striato-limbic and frontal areas. Inter-individual variations in the community-size of these two communities were also associated with the OCI-R score (*p* < .005). Due to our small sample size, we further validated our results by (i) accounting for head motion, (ii) applying global signal regression (GSR) in data processing, and (iii) using an alternate atlas for parcellation. While the main results were consistently observed with accounting for head motion and using another atlas, the key findings were not reproduced with GSR application. The study demonstrated the existence of disconnectedness in fronto-striato-limbic community and connectedness between cerebellar and visual areas in OCD patients, which was also related to the clinical symptomatology of OCD.

## Introduction

Obsessive–compulsive disorder (OCD) is a chronic illness affecting two to three percent of the global population^1,2^. OCD is life-disrupting and is one of the leading causes of major health-economic burdens^3^.

Resting-state functional magnetic resonance imaging (rsfMRI) offers the opportunity to evaluate neural correlates without task influences. Using rsfMRI techniques, prior studies implicate a myriad of neural alterations in patients with OCD, including the basal ganglia, limbic, prefrontal, temporo-parietal, occipital, and cerebellar regions^4–18^. Even though these studies also generally support neural dysfunctions involving the cortico-striato-thalamo-cortical circuitry (CSTC) in OCD pathophysiology, it remains unclear how the whole-brain network organization differs in patients with OCD compared to healthy controls.

The network organization identifies the modular architecture of the brain and reveals how communities are connected to each other^19^. Communities refer to a group of brain areas that are more strongly connected to one another, thereby reflecting the regularities in the brain's wiring diagram, where groups of brain areas get delineated based on the functionalities they share^20,21^. A rsfMRI study that identified group-level differences in network organization between patients with OCD and controls found stronger intra-connectivity between the basal ganglia and cerebellar nodes in patients compared to controls^22^. There is current consensus that individual-based-approaches, when applied to rsfMRI, could better identify neuroimaging biomarkers for OCD^23^.

In our prior work, we developed a technique to decompose the rsfMRI signal into a group-level subspace shared across all subjects within the group, and an individual subspace that is unique to each subject^24^. We showed that the common group-level signal encompassed experimental and environmental (scanner) effects^24–27^, and demonstrated that the individual subspace of subjects could be obtained by removing the group-level signal from the subject’s rsfMRI data. Compared to conventional rsfMRI approaches (where the group-level subspace is typically not removed from the subject’s data), we found that functional connectivity derived using the individual-fMRI-subspace improves the accuracy of functional-connectivity-based predictions of behavioral measures^24^. This was consistent with another study that also reported enhanced accuracy in behavioral score prediction using individual-specific cortical networks^28^. Therefore, in the present study, we utilized individualized approaches in rsfMRI functional connectivity to gain a richer understanding of the neural correlates that are relevant to OCD pathophysiology.

Here we evaluated the benefits of using individual-fMRI-subspace to investigate differences in functional connectivity between patients with OCD and healthy controls. We demonstrated group differences in functional connectivity that were associated with behaviors^29^, by using functional connectivity to predict the scores of some clinical measures^24,28,30–32^. Prediction-based approaches, which are typically applied on large datasets, are thought to augment prediction accuracy of behavioral scores from functional connectivity matrices^33^. Even though our sample is small, we leveraged on the advantages of prediction-based approaches and applied this technique to improve evaluations of group differences using functional connections that were predictive of the clinical scores.

To explore brain organizational differences between the two groups, we detected communities of each OCD and control group using a recently developed multi-subject modularity maximization technique^34^. This technique explores communities in each subject within its own group (i.e., subject-specific community)^34^. The subject-specific communities of all individuals within the group were then combined, forming the group-specific community structure. Group-related differences in community structures could provide interesting clues about the differing neural architecture.

The inter-individual differences in the community structure of a group is a powerful source of behavioral variation^34^. Since neural reorganization may be the impetus to a disorder, association of altered neural network to OCD symptomatology may broaden the avenues for research and treatment. We evaluated the relationships between communities and clinical scores by applying linear regression to investigate the effect of inter-individual variability in the number of nodes in a community on the clinical measure. Understanding inter-individual variability in communities may form the basis for future studies to explore potential brain modulation targets for treatment of OCD^35^.

## Methods

### Overview

Differences in the scores of clinical measures were evaluated between patients with OCD and controls. Preprocessing of the rsfMRI data of all participants was performed to obtain the conventional-fMRI-space. Individual-fMRI-subspace was then obtained from the conventional-fMRI-space. We compared the two spaces by evaluating group differences in functional connectivity. Clinical scores were then predicted from functional connectivity using elastic-net (a machine learning method) to demonstrate that the functional connectivity associated with some behavior in patients with OCD were different from healthy controls. Finally, group differences in brain network organization were evaluated using the community detection technique.

### Participants

The study was granted ethical approval by the Nanyang Technological University Institutional Review Board (NTU-IRB; IRB-2013–11-010) and National Healthcare Group Domain-Specific Review Board (NHG-DSRB; Ref: 2013/01,221). All experiments were performed in accordance with relevant guidelines and regulations with participants’ written informed consent.

Twenty-one patients with OCD (10 males and 11 females) and 22 controls (10 males, 12 females) between the ages of 21–65 participated in the study. Patients with OCD were recruited from the outpatient clinics of the National University Hospital (NUHS) and the Institute of Mental Health (IMH). The healthy controls were age-, sex-, and education-matched to the patient sample and were recruited from the community (see supplement). Data of one female patient with OCD were excluded from the study due to an incomplete resting-state scan. The final dataset included 20 patients with OCD and 22 controls.

All patients only had a diagnosis of OCD without any comorbidities. Out of 20 patients with OCD, all but 2 were on medications at the time of assessment. All 18 patients who were medicated were on serotonin reuptake inhibitors, 3 were on atypical antipsychotics, and 5 on tricyclic antidepressants.

### Procedures

Data collected for the current investigations were part of a larger investigation. Participants completed a series of clinical interviews—Structured Clinical Interview for DSM-IV Axis I Disorders^36^ (Patient/Non-Patient Versions; SCID I-P/NP), Yale-Brown Obsessive–Compulsive Scale^37^ (Y-BOCS), Hamilton Depressive Rating Scale^38^ (HDRS), Hamilton Anxiety Scale^39^ (HAS); and a self-report questionnaire—the Obsessive–Compulsive Inventory-Revised^40^ (OCI-R). All participants underwent an 8-min resting-state scan with eyes opened.

### MRI data acquisition

Images were acquired with a Siemens 3T PRISMA MRI scanner (Siemens Prisma, Erlangen, Germany) using a 32-channel head coil at the Clinical Imaging Research Centre, National University of Singapore. Whole brain MRI structural data were obtained using a high-resolution sagittal T1-weighted 3D MP-RAGE protocol: TR = 1900 ms; TE = 2.52 ms; 176 slices with 1 mm thickness with 0.5 mm gap, flip angle = 9°, the field of view (FOV) = 256 mm, interleaved acquisition. Whole-brain functional MRI images were acquired using gradient-echo planar imaging (EPI) sequence: TR = 2000 ms; TE = 29 ms; flip angle = 90°; 41 slices with 5% gap, voxel size = 3.5 × 3.5 × 3.5 mm; FOV = 225 mm, interleaved acquisition from anterior to posterior. The first five images of the acquisition run were discarded. A total of 240 volumes were obtained for each participant.

### rsfMRI data preprocessing

All neuroimaging data were preprocessed and analyzed using the SPM12 software package (revision #6470)^41^. All images were first reoriented to the AC-PC line with the point of origin set to the anterior commissure. Resting-state functional images were then (i) slice-time corrected to the middle slice^42^, (ii) realigned to the first volume of the run to adjust for head movements, and (iii) co-registered to the T1-weighted image. Individual participant’s structural T1-weighted image was segmented, and the Diffeomorphic Anatomical Registration Through Exponentiated Lie algebra (DARTEL) pipeline^43^ was applied to obtain a group-specific structural template for normalizing of functional images to MNI space, and smoothed with a Gaussian kernel of 6 × 6 x 6 mm full-width at half-maximum (FWHM).

CONN-fMRI Functional Connectivity Toolbox for SPM (version 17b)^44^ was utilized to extract the rsfMRI time-series from 116 regions within the Automated Anatomical Labeling (AAL) atlas that consists of 90 cortical (45 for each hemisphere) and 26 cerebellar regions^45^. To accomplish this, preprocessed data were first temporally band-pass filtered (0.008 < f < 0.09 Hz). White matter and cerebrospinal fluid signals (5 regressors each) and motion parameters (6 motion parameters and their 1^st^ order temporal derivatives) were regressed from the rsfMRI signals using component-based noise correction method (CompCor) implemented in CONN. The CompCor strategy significantly reduces physiological and movement noises without removing the global signal^46^, thereby avoids introducing artifactual anti-correlations^44^. Following CompCor, rsfMRI time-courses were extracted from 116-regions, obtaining a 116 × 240 matrix for each subject with 6 motion parameters included as covariates-of-no-interest (as part of default setting). For convenience, we refer to this as the ‘conventional-fMRI-space’ of a subject.

We computed the mean framewise displacement (FD) as an index of head motion^47^. All participants had mean FD < 0.2 mm. Therefore, motion correction was not performed in the primary analysis. Global signal regression (GSR) was also not performed in the primary analysis as GSR introduces artifactual anti-correlations and spurious findings in comparison-based studies^48^. However, we recognized that motion correction and GSR procedures are commonly performed in functional connectivity analyses. In the later parts of the paper, we validated our findings by accounting for head motion using FD as a covariate and applied GSR separately in additional analyses (please see the *validation* section).

### Individual-fMRI-subspace

The algorithm to obtain individual-fMRI-subspace for each subject is presented in greater detail in prior works^24,49,50^. Briefly, the conventional-fMRI-space of a subject was taken as a block. The conventional-fMRI-space (116 × 240 matrix) of all subjects within each group was stacked, forming two sets of multi-blocks (OCD, controls). The group-level common component (116 × C matrix) shared by all subjects within each set was then computed and removed from the conventional-fMRI-space of each subject to obtain the individual-fMRI-subspace (116 × 240 matrix). The number of group-level common component C was fixed to 1 based on a past recommendation^50^, which was also appropriate as demonstrated in our prior study^24^.

### Functional connectivity

We computed a 116 × 116 Pearson’s correlation matrix for all subjects in both groups based on the resting state data from (1) conventional-fMRI-space, and (2) individual-fMRI-subspace. In each space, functional connectivity values (edge strength) that were significantly different between OCD and control groups were extracted at false discovery rate (FDR) < .05 to account for multiple comparisons. The total number of significant connections whose edge strengths were different between the two groups was extracted from both conventional-fMRI-space and individual-fMRI-subspace.

### Assessment of behavior-connectivity associations

Behavior-connectivity association was assessed for clinical measures (state anxiety severity, and obsessive–compulsive tendencies) that were significantly different (*p* < .05, following Bonferroni-corrections) between patients with OCD and controls. For each measure, the elastic-net^51,52^ algorithm was used to predict the subject's clinical score^24^ in a leave-one-out nested cross-validation scheme using the functional connectivity matrices obtained from individual-fMRI-subspace only (see results). Specifically, data from *n* − 1 participants were used to train the model, and the resulting trained model was then applied to predict the remaining participant’s score of clinical measure. Age, sex, and years of education were regressed from the measures before elastic-net regression. The nuisance regression was performed on the training folds, and the estimated coefficients were then applied to the test fold.

After nuisance regression, the training folds were used for feature selection by selecting the top 50% of functional connections that were most strongly correlated (positive or negative) with the particular clinical measure within each group^24^. The selected features (functional connectivity strength) were then entered into the elastic-net regression estimation procedure. Two hyperparameters associated with the elastic-net were first optimized via leave-one-out inner-loop cross-validation, then applied to predict the clinical measure in the test fold. Accuracy in prediction was evaluated by correlating the predicted and actual values of the clinical measures across the subject in the test fold within each group^30^. The prediction *R*^*2*^ was also computed to directly compare the predicted and observed values^53,54^. Prediction results obtained from training and testing on the OCD dataset were referred to as ‘Train-OCD-Test-OCD’, and controls dataset as ‘Train-Control-Test-Control’. We evaluated whether functional connections that were relevant in predicting the clinical scores were different between the groups. For this purpose, we created random subgroups of controls (RSC) and patients with OCD (RSO) using bootstrapping (see next paragraph). We trained the algorithm on RSC and tested the algorithm on a subject randomly chosen from the OCD group (we refer to this as ‘Train-Control-Test-OCD’), and vice-versa—trained the algorithm using random subgroups of OCD subjects (RSO) and tested it on a randomly chosen control (we refer to this as ‘Train-OCD-Test-Control’). Prediction indices (accuracy and prediction *R*^*2*^) were also computed in this cross-dimensional approach.

To statistically evaluate the prediction accuracies between Train-OCD-Test-OCD and Train-Control-Test-Control from Train-Control-Test-OCD and Train-OCD-Test-Control, respectively, we first applied the leave-one-out bootstrap technique^55^ to the four groups. The leave-one-out bootstrap procedure generates a total of *B* bootstrap samples of size *n*. Using the prediction model (elastic-net in leave-one-out scheme), each observed specimen is predicted repeatedly using the bootstrap samples such that the test observation does not appear in training. This technique is particularly suitable for small sample sizes^56^. We chose *B* = 1000 based on previous literature^57^, and estimated the prediction accuracy and prediction *R*^*2*^. For these two groups (Train-OCD-Test-OCD and Train-Control-Test-Control), a one-sample *t* test was performed on the prediction accuracy values of the permuted samples to test whether, for each behavioral measure, prediction accuracy was significantly greater than chance. For Train-Control-Test-OCD and Train-OCD-Test-Control, we adopted an approach to avoid leakage of data from training to testing samples^58^. For each bootstrapped training sample, the prediction was made on the test data (chosen from the other dataset) selected randomly (but not repeated for a bootstrapped test sample). The prediction accuracy and prediction *R*^*2*^ were eventually obtained for each model. To test for significance, a two-tailed *t* test with FDR-correction (*p* < .05) was performed on the prediction accuracies between Train-OCD-Test-OCD & Train-Control-Test-OCD, and Train-Control-Test-Control & Train-OCD-Test-Control. At this point, it is essential to mention that our study was not intended to emphasize the predictive accuracy of small sample sizes^58^, although prior studies have made such attempts^59^. We planned to use the prediction-based approach as a tool to highlight differences in the functional neural architecture of the two groups.

Next, we estimated the total number of functional connections that were common between OCD patients and controls for each bootstrapped sample using the approach validated in a previous study^23^. In this evaluation the weight of each functional connection used for behavior prediction was computed as the averaged-weight across all leave-one-out bootstrap folds. If a connection was not selected in one-fold, its weight was set to zero. Hence, each connection obtained weights in the range of zero to N − 1, where N was the number of folds. The higher the weight, the greater the plausibility of the connection contributing to behavior-connectivity associations. To evaluate the overlap in functional connectivity architecture between the two groups, we applied three threshold conditions by selecting the top- (A) 20%, (B) 50%, and (C) 100% weighted connections of each group. As no threshold was applied in the 100% condition, all weighted connections of both groups were selected for evaluation. For each threshold condition, similarities in weighted connections between the two groups were estimated using the Jaccard index. This was computed by dividing the number of weighted connections that were common between the two groups (intersecting) by the total number of weighted connections (union). Connections were considered common when the same connection was selected for evaluation in both groups. The Jaccard index ranges between 0 and 1. A value of 0 indicates no overlap between the groups, and a maximal value of 1 indicates perfect overlap. A one-tailed *t*-test was done on values of the Jaccard index obtained from the permuted samples to test for a significant overlap.

### Community detection

To detect communities, we adapted a multi-layer multi-subject modularity maximization framework^34^ that was based on the Louvain method for community detection^60,61^. Here, we briefly describe this framework that facilitated the analysis of inter-individual differences in network communities.

A subject’s functional connectivity matrix was treated as a layer. Therefore, multiple layers were used to estimate the community assignment for all subjects. Importantly, there are two free parameters, namely the structural resolution parameter γ that determines the community size, and the inter-subject coupling parameter ω that determines the consistency of communities across layers. Smaller values of γ result in fewer large communities, and larger values ω indicate communities that are common across subjects. One other important parameter is the entropy that determines the variability of the communities across subjects. The averaged normalized entropy serves as an index of community variability across brain areas and individuals. This framework is typically applied on a restricted subset of the {γ,ω} parameter space, so that communities have characteristic size and variability.

Here, we sampled 20,000 partitions in the parameter space {γ,ω} with γ and ω acquired in the range of [− 0.7, 1] and [− 0.5, 2], respectively^34^. We obtained the *mean of the averaged-normalized-entropy* (MANE) across all the partitions of each group. To search for the optimal community partition, we explored the community partitions whose averaged normalized entropy lies within the range of MANE ± 0.02. To select the optimal community partition for the purpose of our study, we chose the partition where (1) value of γ is minimum (so that few large communities appear), (2) value of ω is maximum (to ensure that communities are shared across subjects within the group), and (3) community assignment did not show any community that contain a single node in any of the subjects (in that partition)^34^. In addition, prior studies that delineated several large-scale networks using rsfMRI aided our decision regarding appropriate community-level partitions and naming convention^62,63^ with a range from 5 to 23 communities/networks as widely reported^20,64–66^.

#### Association of communities with behavioral measures

After selecting the optimal community partition, we obtained the inter-individual variation in the number of nodes assigned to a community. The extent to which inter-individual variation in the community size is associated with any clinical measure was explored using linear regression via Matlab's “fitlm.” function. The model was constructed as behavior ~ inter-individual variation of nodes in Community 1 + inter-individual variation of nodes in Community 2 + … + inter-individual variation of nodes in Community N (where N is the optimal number of communities in a group).

As patients with OCD showed increased depressive and anxiety symptoms compared to controls, we included scores from HDRS and HAM-A as covariates in the model for the OCI-R measure for both groups. Therefore, the model was: behavior ~ inter-individual variation of nodes in Community 1 + inter-individual variation of nodes in Community 2 + … + inter-individual variation of nodes in Community N + scores of HAM-A + scores of HDRS.

### Additional analysis for validation

To further validate our findings, we conducted the following additional analyses by (i) accounting for the effects of head motion, (ii) applying global signal regression in data preprocessing, and (iii) using an alternate atlas for parcellation.

#### (i) Accounting for effects of head motion

As head motion could influence functional connectivity^67^ and prediction accuracy^68^, we evaluated whether functional connections that were associated with predicting clinical scores remained after correcting for head motion. Head motion of each participant was indexed using mean FD (Mean = 0.074 mm, *SD* = 0.040 mm)^47^. The rsfMRI time-courses were extracted with each subject’s mean FD included as a covariate-of-no-interest. Next, we estimated group differences in functional connectivity (differences in edge strength). For the elastic-net, we regressed out mean FD along with age, sex, and education. The remaining steps such as training and testing the algorithm, permuting new samples and estimating the relevant functional connections remained the same. For convenience, we called this set of data ‘Train-OCD-Test-OCD (FD-Corrected)’ and ‘Train-Control-Test-Control (FD-Corrected)’. A two-tailed *t* test with *p* < .05 FDR-correction was performed to assess for group differences in prediction accuracies between Train-OCD-Test-OCD and Train-OCD-Test-OCD (FD-Corrected) and between Train-Control-Test-Control and Train-Control-Test-Control (FD-Corrected) datasets. Next, we tested for group differences in the associations between motion (mean FD) and edges. For this purpose, we correlated the mean FD with the strength of edges. The values were then converted to Fisher’s *z* and a two-tailed *t* test (with FDR-correction) was performed to evaluate group differences. Finally, we tested if the communities were altered when head motion was accounted for.

#### (ii) Applying global signal regression in data processing

There is much debate on the use of GSR in preprocessing. While GSR reduces the impact of motion^67,68^ and strengthens brain-behavior associations^69^, GSR introduces negative correlations^48,70,71^ that could influence group differences^72,73^. We repeated our main analyses to understand the impact of GSR on functional connections that were associated with predicting clinical scores.

Following band-pass filtering in CONN, instead of adopting the CompCor strategy for noise-reduction, we conducted GSR noise reduction by including global signal (1 regressor) and motion parameters (6 motion parameters and their 1^st^ order temporal derivatives). Subsequent steps were the same as the primary analyses. Following noise reduction, rsfMRI time-courses were extracted with 6 motion parameters included as covariates-of-no-interest. We applied the same procedure of permuting the new samples and estimating the relevant functional connections. For convenience, we call this set of data ‘Train-OCD-Test-OCD (GSR)’ and ‘Train-Control-Test-Control (GSR).’ A two-tailed *t* test was performed to assess for group differences in prediction accuracies between Train-OCD-Test-OCD and Train-OCD-Test-OCD (GSR), and between Train-Control-Test-Control and Train-Control-Test-Control (GSR). Lastly, the impact of GSR on community structures was evaluated.

#### (iii) Using an alternate atlas for parcellation

Our choice of the atlas was based on the consideration to include both cortical and subcortical region of interests (ROIs). However, due to our small sample size, we refrained from using fine-grained parcellations (for example, Schaefer^74^, Glasser^75^) that comprise a large number of ROIs. While we initially utilized the AAL atlas for parcellation, we validated our findings by repeating our analyses on the FD-corrected data using the Harvard–Oxford atlas. The Harvard–Oxford atlas consists of 132 cortical and subcortical areas. In this analysis, we focused on community detection (with predefined criteria of community selection) and deciphering the associations between inter-individual variation in the nodes of each community and clinical measures (HAM-A and OCI-R).

### Code availability

The codes relevant to the study can be downloaded at (https://github.com/ClinicalBrainLab/OCD_Cerebellar-Visual-Community). The codes for elastic-net are available at https://web.stanford.edu/~hastie/glmnet_matlab/. The code for multi-subject community detection is at https://www.brainnetworkslab.com/coderesources.

## Results

### Overview

Patients with OCD and controls showed group differences in two clinical measures. We found using the individual-fMRI-subspace advantageous in evaluating group differences in functional connectivity. Behavior-connectivity association analysis indicated group differences in functional connectivity architecture that were associated with clinical measures. Finally, in terms of functional organization, patients with OCD showed connectivity between the cerebellar regions with visual nodes and lack of connectivity between the striato-limbic and frontal areas that were not observed in controls.

### Demographics and clinical measures

There were no significant group differences in demographic variables (Table 1). Compared to healthy controls, patients with OCD reported higher anxiety scores and OC scores as measured on the HAM-A and OCI-R, respectively (*p* < .05, following Bonferroni-corrections).

**Table 1:** Descriptives of demographics and clinical indices.

Gender (male:female) (count)	Controls			OCD			χ^2^	*df*	*p*	Effect size
	10:12			10:10			0.09	1	.77	
	Controls (*n* = 22)			OCD (*n* = 20)			Mann–Whitney			
	*Mdn*	*M*	*SD*	*Mdn*	*M*	*SD*	*U*	*p*	*z*	*r*
**Demographics**										
Age	27.0	28.18	6.66	29	28.80	7.02	206.00	.72	–	–
Years of education	13.0	14.41	2.06	14.5	14.55	2.19	202.50	.63	–	–
Handedness Index^a^	100.0	79.09	36.19	100	84.15	44.61	185.00	.31	–	–
**Clinical indices**										
HDRS	2.0	2.55	1.82	4	5.30	4.61	134.00	.03	− 2.18	− .34
HAM-A	2.5	2.50	1.68	7	7.05	5.91	107.50	.004*	− 2.85	− .44
OCI-R	2.5	3.45	3.33	21	23.20	15.47	18.50	< .001*	− 5.09	− .79
Duration of illness (years)	–	–	–	–	8.18	6.59				
Y-BOCS (total)	–	–	–	–	19.90	6.61	–	–	–	–
Y-BOCS (obsessions)	–	–	–	–	9.70	3.34	–	–	–	–
Y-BOCS (compulsions)	–	–	–	–	10.20	3.87	–	–		

^a^Handedness index is measured using Edinburgh's Handedness Questionnaire.

*Indicates findings significant at .05 following Bonferroni-correction.

*HDRS* Hamilton depressive rating scale, *HAM-A* Hamilton anxiety rating scale, *OCI-R* obsessive–compulsive inventory (revised), *Y-BOCS* Yale–Brown obsessive compulsive scale. *n* sample size. *Mdn* median, *M* mean, *SD* standard deviation, *r* effect size coefficient, where .1 = small, .3 = medium, .5 = large effects.

### Spatial maps of common components

**Figure 1:**
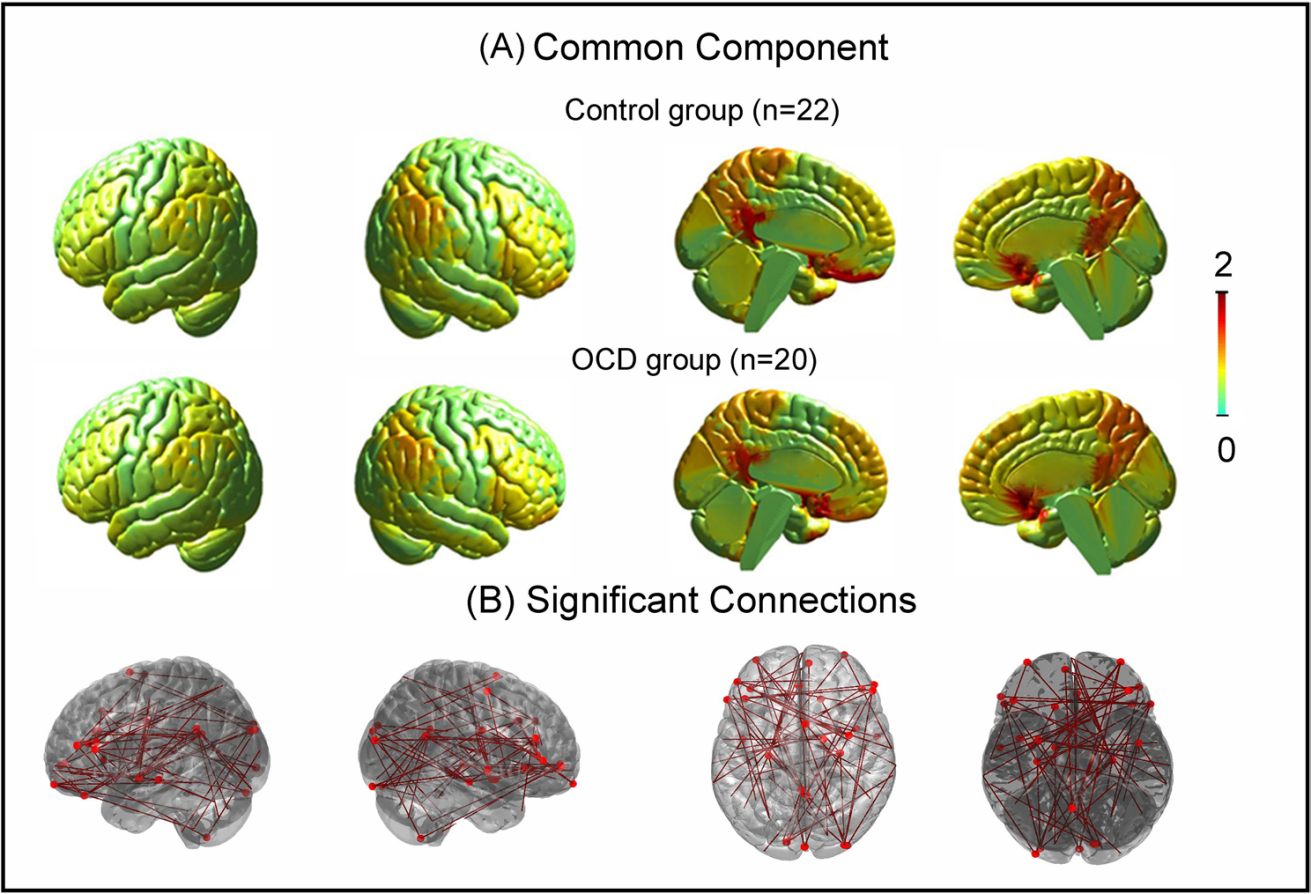
Represents the features and advantage of individual-fMRI-space with (**A**) spatial map of the common component for the control and OCD group. Maps are similar in both groups and also similar to our previous study^24^, (**B**) significant differences in 83 functional connections between patients with OCD and control. The connections survived FDR-corrected *p* < .05. No group difference in functional connectivity was observed from using conventional-fMRI (therefore not shown).

Spatial maps of the first common component were not significantly different for patients with OCD and healthy controls (*p* > .05) (Fig. 1A).

### Functional connectivity differences from conventional and individual-fMRI-spaces

Functional connectivity differences between the OCD samples and controls using the conventional-fMRI-space did not find any connections that survived FDR-correction. In contrast, the same analysis conducted using individual-fMRI-subspace yielded significant group differences in 83 functional connections, *p* < .05, FDR-corrected (Fig. 1B). To confirm that the edges were indeed significant, we performed 1000 iterations of permutation testing (shuffling subject labels), and used the "tmax" method to account for multiple comparisons at a particular threshold (α-level) ^76^. We found that 56 edges (or connections) were significantly different between the groups at α = .05, and this number decreased to 14 when a more stringent threshold of α = .01 was applied. The small differences in the number of edges revealed using each method suggests that the findings were considerably stable.

Although not the main focus of the study, we briefly present the group differences in functional connectivity here. Group differences appear distributed (Fig. 1B), as was expected based on the findings reported from previous studies^4, 17, 18, 77^. Connections were observed in several subcortical, cortical, and cerebellar regions, including areas in the frontal lobe, cingulate, cuneus, limbic system (hippocampus, parahippocampus, amygdala, thalamus, olfactory, and insula), visual areas (extrastriate and lingual gyrus), sensorimotor (pre- and post-central, inferior- and superior- parietal, and supramarginal gyrus), temporal regions (superior, middle and heschl gyrus), and the cerebellum (lobules 4–6, 9 and 10). Connections between these areas encompassed several brain networks, including the frontal-limbic-posterior circuit, fronto-striatal, fronto-parietal, and cerebellar-visual circuits, which were reported in prior studies (albeit with inconsistencies)^2, 5–7, 9, 11^ and consolidated in review-based studies^4, 78^ (Fig. 1).

### Differences in functional architecture between OCD and controls

For the complete samples, we estimated the prediction accuracy (and prediction *R*^*2*^) of the two models, namely Train-Control-Test-Control (*n* = 22) and Train-OCD-Test-OCD (*n* = 20) for the two clinical measures (HAM-A and OCI-R) that showed significant group differences. For HAM-A, the prediction accuracy (and prediction *R*^*2*^) for Train-Control-Test-Control and Train-OCD-Test-OCD were 0.71 (0.48) and 0.65 (0.40), respectively. Similarly, for OCI-R, the values for Train-Control-Test-Control and Train-OCD-Test-OCD were 0.67 (0.42) and 0.63 (0.40), respectively.

For the bootstrapped samples, the prediction accuracy (and prediction *R*^*2*^) were estimated for the four models listed in Table 2 for HAM-A and OCI-R. Results showed that prediction values for the measures were higher when training and testing were performed on data belonging to the same group (Train-Control-Test-Control and Train-OCD-Test-OCD). One sample *t-*test was significant (*p* < .0001) for both groups across the two measures. Please refer to the supplement for the same analyses conducted for the HDRS and Y-BOCS (Total).

**Table 2:** Average (± *SD*) leave-one-out bootstrap prediction accuracy and prediction *R*^*2*^ (in brackets and bolded) for the two clinical measures estimated across 1000 permutations.

Clinical measure	Average (± *SD*) leave-one-out bootstrap prediction accuracy and prediction R^2^			
	Train-control- Test-control	Train-OCD- Test-OCD	Train-control- Test-OCD	Train-OCD- Test-control
HAM-A	0.69 ± 0.20	0.68 ± 0.20	0.17 ± 0.16	0.12 ± 0.19
	(**0.45 ± 0.31**)	(**0.43 ± 0.32**)	(**0.02 ± 0.10**)	(**0.01 ± .12**)
OCI-R	0.68 ± 0.23	0.66 ± 0.22	0.08 ± 0.24	0.06 ± 0.27
	(**0.42 ± 0.31**)	(**0.40 ± 0.29**)	**(0.00 ± 0.10)**	(**0.00 ± 0.11**)

Prediction *R*^*2*^ is indicated in bold within parentheses. *HAM-A* Hamilton Anxiety Rating Scale, *OCI-R* Obsessive–Compulsive Inventory (Revised), *SD* standard deviation.

The accuracies (and prediction *R*^*2*^) were reduced when training and testing data were obtained from different groups (Train-Control-Test-OCD and Train-OCD-Test-Control). Significantly (*p* < .05, FDR-corrected) lower values of prediction accuracies were found for Train-Control-Test-OCD and Train-OCD-Test-Control compared to Train-Control-Test-Control and Train-OCD-Test-Control in the permuted samples, respectively. This indicates that the functional connections recruited during training were different from that required to have high prediction accuracy in the testing phase. The analysis supports our hypothesis that there exist differences in functional connectivity architecture in patients with OCD and controls. While our study does not make any recommendations for applying prediction-based approaches to datasets with small sample sizes, we leveraged on the advantages of these techniques to maximize differences in the functional organization between two groups.

Group differences in the functional connections that were associated with the two clinical measures were further demonstrated using the Jaccard index ($$\frac{Number of common connections}{Total number of connections} ).$$ The Jaccard index (Mean ± *SD*) calculated from bootstrapped samples for the three threshold conditions is shown in Table 3. Values near 0 indicate that the number of common weighted connections between the OCD group and the controls is low. The results showed almost no overlap of connections between the two groups in the Top 20% condition (JI values were almost 0) and remains minimal in the 50% condition. The overlap in weighted connections at no-threshold condition (Top 100%) may look larger (approximately 100), but less than one-tenth (approximately) of the total connections were common between the two groups. This indicates that only 10% of the unweighted connections were common between the two groups. The lower values of the Jaccard index (approximately 0.05–0.08) for the two clinical measures affirms the minimal overlap. Please refer to the supplement for the values estimated for HDRS and Y-BOCS (Total).

**Table 3:** Overlap (Mean ± *SD*) of weighted connections between OCD patients and controls estimated across the bootstrapped samples for three threshold conditions.

Clinical Measure	Top 20% threshold				Top 50% threshold				No-threshold (top 100%)			
	Controls	OCD	OL	JI	Controls	OCD	OL	JI	Controls	OCD	OL	JI
HAM-A	9 ± 20	11 ± 16	0 ± 1	0.05 ± 0.07	128 ± 82	154 ± 76	8 ± 18	0.03 ± 0.06	730 ± 476	1030 ± 520	98 ± 87	0.05 ± 0.10
OCI-R	3 ± 17	10 ± 21	0 ± 3	0.00 ± 0.12	212 ± 56	205 ± 110	16 ± 11	0.05 ± 0.07	687 ± 547	1201 ± 396	106 ± 93	0.08 ± 0.12

Results were only presented for HAM-A and OCI-R. The number of overlaps (OL) is negligible compared to the total number of connections, as reflected by smaller values of the Jaccard index (JI). Each connection was given a weight based on the number of times it appeared in the leave-one-out bootstrap scheme for that group. Thresholding (as specified) was performed to select the connections that contributed most to the prediction. The overlap represents the number of connections that were common between the two groups. *HAM-A* Hamilton Anxiety Rating Scale, *OCI-R* Obsessive–Compulsive Inventory (Revised), *SD* standard deviation, *OL* overlap, *JI* Jaccard index.

It may be observed that 83 edges that were different between OCD and controls constitute significance in the earlier part of the study, but here, we concluded that approximately 100 overlapping edges does not constitute significant effect in the prediction architecture. To evaluate this finding, we estimated the overlap between the edges that ‘overlapped-between-two-groups’ (in each permutation) and the ‘83 connections that differentiated the two groups’. For HAM-A and OCI-R, the range of the overlap was from 15 − 33% and 18 − 38%, respectively. This suggests that, although the 83 functional connections that differed between the two groups were relevant in differentiating them, the same connections may be inherent in the networks for both groups related to a behaviour that is on a continuum as reflected in the clinical scores. Future research could evaluate how connections that differ between groups may inform about the relevant dimensions of behavior(s) involved. Nevertheless, using the prediction-based strategy augmented our understanding that there exists divergence in the functional architecture between the two groups. This provides evidence regarding differences in the underlying organization of the brain between patients with OCD and controls.

Group differences in the predictors (i.e., functional connections as seen in Table 2) indicate different functional connections were associated with state anxiety and obsessive–compulsive tendencies in patients with OCD compared to controls. A similar observation was also seen in Table 3. Taken together, these findings indicate that the functional organization in healthy individuals is different from patients with OCD.

### Differences in community assignment in OCD and controls

**Figure 2:**
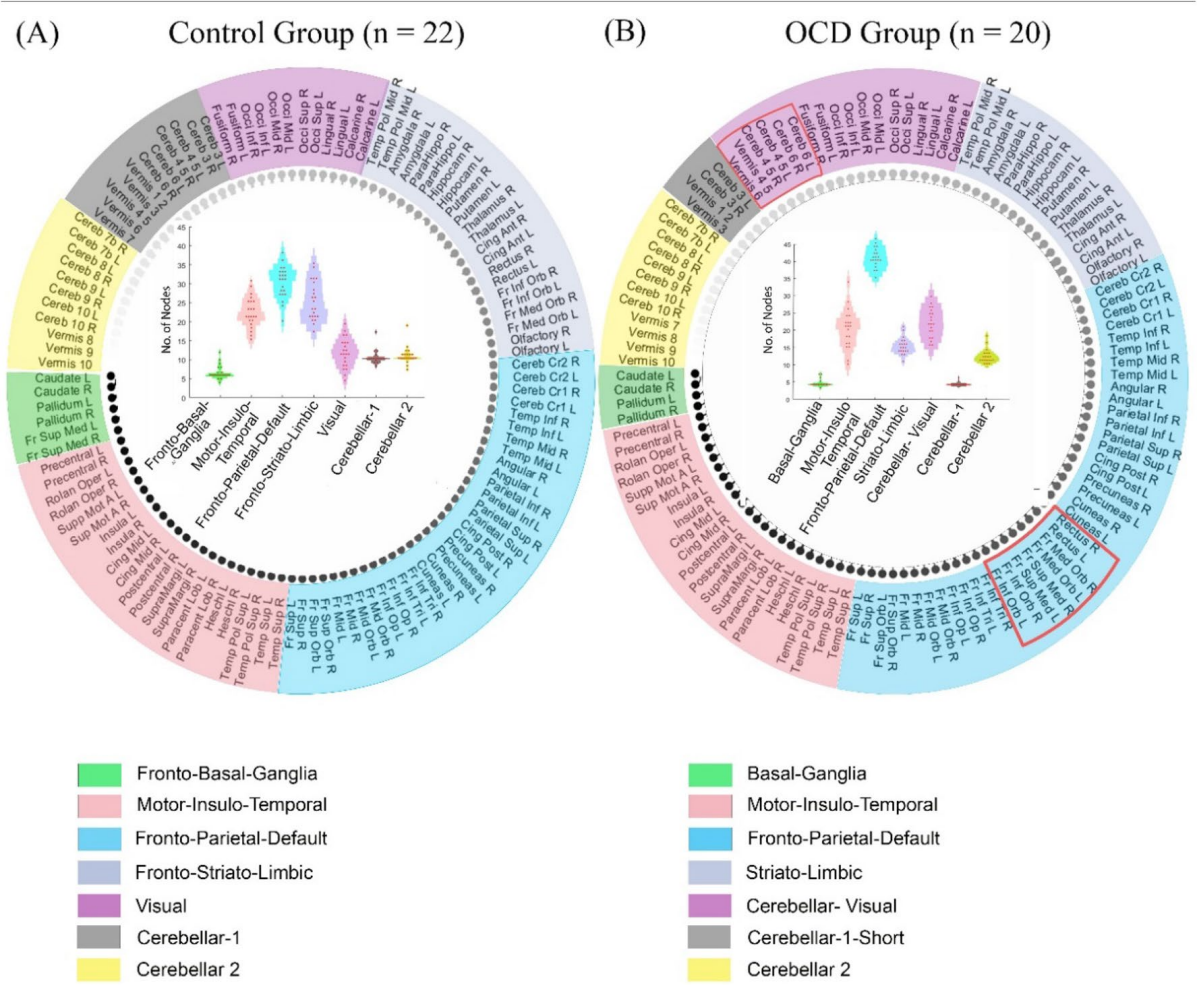
Communities in each control and patient group. The outer circle shows the median of the number of nodes (in a community) across the subjects and brain areas involved in each community. The communities (colored regions) are named based on the areas they encompass. Inside the inner circle, the inter-individual variation (red dots inside the violin plots, where one red dot = one participant) in the number of nodes in each community can be visualized for the control group. Seven communities were delineated for the (**A**) controls, and (**B**) patients with OCD. We also highlight that the frontal and cerebellar areas were observed only in the OCD group but not control group (enclosed in red boundaries). The abbreviations of brain areas were labelled according to the AAL atlas (please refer to the supplement for a full expansion of the abbreviations).

We estimated the underlying network organization of the brain in each group using community detection technique^34^. Differences in network organization are vital in understanding the neural architecture in OCD. The values obtained with the predefined criteria for community selection (see supplement) revealed seven communities as optimal for both OCD and Control groups (Fig. 2). For convenience, we assigned a name to the communities for each group based on the areas they encompassed (Supplement). The community names for the control group (Fig. 2A) were fronto-basal-ganglia, motor-insulo-temporal, fronto-parietal-default, fronto-striato-limbic, visual, cerebellar-1, and cerebellar-2. The community names for the OCD group (Fig. 2B) were basal-ganglia, motor-insulo-temporal, fronto-parietal-default, striato-limbic, cerebellar-visual, cerebellar-1-short, and cerebellar-2. Differences in communities were observed between the groups. Interestingly in the OCD group, the areas in the frontal lobe dissociated from the striatal, limbic, and basal ganglia regions; areas in the cerebellum were associated with the visual areas, which were not observed in the controls group.

The number of nodes of each community in a subject within their group (OCD and control) was estimated. The inter-individual variation in the number of nodes in each community (or community size) can be visualized for the control and OCD groups inside the inner circle of Figs. 2A,B, respectively.

### Associations of communities with behavioral measures

We evaluated the associations of inter-individual variation in community sizes (number of functional connections) with the two clinical measures (HAM-A and OCI-R). The model was significant (*p* < .01) only for the OCI-R scores in both groups (for details see supplement). Since the sample size is small, we performed post-hoc power analysis and found 83% power at α = .05 to detect effect size with Cohen’s f^2^ = 1.31^79^. Inter-individual variations in the community size were found significant for two communities (fronto-parieto-default and fronto-striato-limbic) in the control group, but three communities (fronto-parieto-default, striato-limbic, and cerebellar-visual) in the patient group (*p* < .05, FDR-corrected). For each group, we correlated the values of OCI-R with the scores of HAM-A and HDRS. For controls, the correlation between OCI-R and HAM-A was .41, and OCI-R and HDRS were .32. Similarly, for the OCD group, the correlation between OCI-R and HAM-A was .75, and OCI-R and HDRS were .80. As depression and anxiety scores were highly correlated with OCI-R scores, we included these scores as covariates-of-no-interest. Interestingly, we found that for the OCI-R measure, the model was no longer significant for the OCD group but remained significant (*p* < .01) for the controls.

### Validations

We validated our results by conducting additional analyses by (i) accounting for the effects of head motion, (ii) applying global signal regression in data preprocessing, and (iii) using an alternate atlas for parcellation.

#### (i) Accounting for effects of head motion

**Figure 3:**
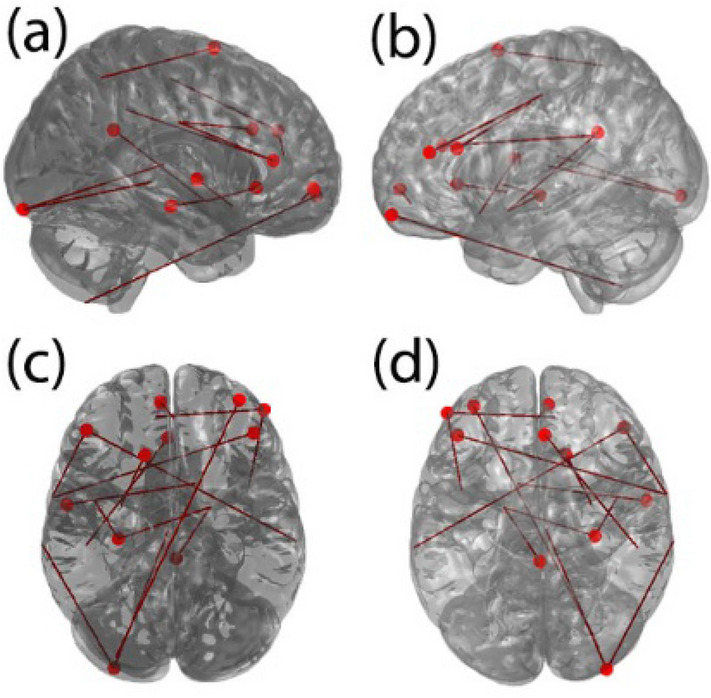
Shows the effect of motion correction on the functional connectivity differences between the two groups. As in Fig. 1, 83 functional connections were significantly different between the two groups prior to motion correction. However, only 76 connections were different between the two groups after motion correction. There was a large overlap between the two sets of functional connections (83 and 76), and only 15 connections are different between the two sets of data (‘without’ versus ‘with’ motion correction). The location of the nodes and the edges of these 15 functional connections are shown across the four different views (a, b, c, and d) of the brain.

Group differences in mean FD were significant (*p* < .01) between patients with OCD and controls. Differences in functional connectivity controlling for mean FD revealed 76 connections that were significantly different between the two groups. However, using permutation testing at α = .05, this number dropped to 53 connections, and further dropped to 11 when the α-level increased to .01. To understand which connections were affected by motion correction, we estimated the overlap between the connections that were significantly different between the two groups — ‘with’ (76 connection) and ‘without’ (83 connections) accounting for head motion. Only 15 connections were different between the two datasets (Fig. 3). Thus, accounting for head motion may minimize false positives in the group differences in functional connectivity.

Interestingly, the features (functional connections) recruited for predictions were not significantly different between Train-OCD-Test-OCD and Train-OCD-Test-OCD (FD Corrected); and between Train-Control-Test-Control and Train-Control-Test-Control (FD Corrected), all *p* > .05. There were also no significant group differences in the associations between mean FD and edge strength. Finally, the community structure remained similar to our primary findings, and the inter-individual variation in the number of nodes in a community was significant (*p* < .01) for OCI-R. Three communities (fronto-parieto-default, striato-limbic and cerebellar-visual) showed significant inter-individual variation within the OCD group, whereas only two communities (fronto-parieto-default and fronto-striato-limbic) were significant in controls (*p* < .05, FDR-corrected). In general, the results observed from our primary analyses remained even when head motion was accounted for.

#### (ii) Applying global signal regression in data processing

Following GSR application in data processing, the number of functional connections that significantly differed between the two groups dropped from the initial 83–54 connections, and even further, to 18 connections with permutation testing using “tmax” at α = .01. Primary findings, such as the connections between the cerebellum and other areas (visual and frontal) were no longer significant with GSR application. Features (functional connections) that were recruited for predictions were significantly different (*p* < .05) between Train-OCD-Test-OCD and Train-OCD-Test-OCD (GSR); and between Train-Control-Test-Control and Train-Control-Test-Control (GSR). The Cerebellar-Visual community that were previously observed in the OCD group were no longer observed.

#### (iii) Using an alternate atlas for parcellation

Six communities were detected for each control and OCD group using the Harvard–Oxford atlas that comprises 132 ROIs (supplement) compared to seven communities that were reported in the primary analyses using the AAL atlas. This discrepancy was expected as the areas were specified differently in the two atlases, therefore, leading to different community structures. Nevertheless, the disconnection of the striato-limbic regions from frontal regions, as well as alliance of the cerebellar regions with visual regions were still observed in the OCD group using the Harvard–Oxford atlas. Associations of the inter-individual variation in community size with the two clinical measures were assessed, and only the model for the OCI-R score was significant (*p* < .001) in both groups. Inter-individual variation in the community size were significant in two communities in both groups—the default mode and cerebellar-visual communities within the OCD group, and the default mode and fronto-striato-limbic communities in the control group (*p* < .05, FDR-corrected). The use of a different atlas did not alter the interpretation of our key study findings.

## Discussion

In this study, we sought to clarify the pathophysiology of OCD using individualized approaches. In general, functional connectivity derived from individual-fMRI-subspace appear to have an edge over conventional-fMRI-space in revealing functional connectivity differences between patients with OCD and controls.

The rsfMRI signals can be decomposed into a group-level subspace that is shared across all subjects within the group, and an individual subspace that is unique to each subject^24^. We extracted the spatial maps of the common group-level signal within each OCD and control group, and showed that, while the common group-level signal (specific to each group) appear similar, they were not identical (see supplement). Consistent with prior observations^24^, the common group-level signal observed in our study included regions such as the posterior cingulate areas and prefrontal cortex. These regions were reportedly involved in monitoring the scanner environment and scanner noise^25–27^. The presence of this common group-level signal in fMRI data, as with the conventional-fMRI-space, reduces inter-individual differences of subjects within the group, thereby, decreasing individual distinctiveness in the data. The individual-fMRI-subspace can be extracted by removing the common group-level signal from the individual’s conventional-fMRI-space. A more in-depth discussion on the importance of removing the common group-level signal to obtain the individual-fMRI-subspace is discussed in our previous work^24^.

Considering that the common group-level signal in both groups appear similar, instead of extracting the common group-level signal that is specific to each group, one may be tempted to first combine the two groups, then extract the common component to obtain individual fMRI subspace of each subject. We do not recommend this approach, as doing so may result in erroneous removal of signals that may not be “common” to the specific group (OCD / Controls). We provided further explanations in the supplement.

Here, we found that using individual-fMRI-subspaces in functional connectivity analyses improved the detection of differences between OCD patients and controls (Fig. 1B). While no group differences in functional connectivity were observed using the conventional-fMRI-space approach, 83 connections between the nodes in the frontal cortex, temporo-parietal, limbic, and cerebellar regions were observed to be different between the groups using the individual-fMRI-subspaces approach. These connections were previously reported in various studies^4,9,10,14,22,77,80^. Thus, analyses using the individual-fMRI-subspace approach may offer an opportunity to deepen our understanding of neural alterations in patients with OCD.

To further understand the underlying differences between the groups, we used the functional connectivity derived from individual-fMRI-subspace to map the differences in functional connectivity architecture of patients with OCD and controls. This is achieved by applying the widely-used connectome-based-predictive modeling^28,30–32,81,82^ in our study. We established that the functional connections recruited by OCD patients were different from those of controls (Tables 2 and 3). The significant drop in the prediction accuracy when we trained our predictive algorithm on a dataset (OCD/control) but tested the prediction accuracy on a different dataset (control/OCD) indicated specificity in the functional connections within each group (Table 2). Together, our findings suggest that the functional architecture is different between patients with OCD compared to controls.

In terms of network organization, seven communities were obtained in each OCD and control group. The network organization in OCD patients and controls were similar in three communities: motor-insulo-temporal, fronto-parietal-default, and cerebellar, and different in four communities. Differences in the communities between OCD patients and controls were contributed by disconnection of the frontal areas from limbic and striatal regions, and connection of the cerebellar regions with the visual system in the OCD sample. Altered network organization have been observed in neurological disorders^83,84^, but understudied in OCD. The fronto-striato-limbic community that is observed in controls but not in the OCD group, is a major component of the CSTC, and has been implicated in studies investigating OCD^4,11,78^. There is also an increase in recent observations involving the cerebellum and visual regions in OCD pathophysiology^18,85,86^. It may be possible that the connectivity of cerebellar and visual regions may compensate for fronto-striato-limbic dissociations^87,88^. Nakao and colleagues^78^ reviewed a vast collection of OCD research and proposed the OCD circuity be extended beyond the fronto-striato-limbic circuit with inclusion of the occipital lobe and cerebellum that might be involved in processing the visuospatial information. Our findings appear to support their proposal of an extended circuitry.

The OCI-R is a reliable and well-validated scale that differentiates between patients with OCD and controls^40^. Our data showed that compared to controls, patients with OCD reported greater distress associated with their OC symptoms. We showed that within OCD patients, OCI-R tends to associate significantly with inter-individual variation in community size of fronto-parieto-default, striato-limbic, and cerebellar-visual, but with fronto-parieto-default and fronto-striato-limbic brain networks in controls. Compared to controls where frontal regions appear to be functionally connected with limbic areas, patients with OCD showed similar communities but without the communities involving the frontal areas. Drawing reference from what is known about the CSTC circuitry^89^, where the frontal regions help regulate excitatory signals that were elicited from the striatum, the lack of regulatory connections in the striato-limbic community in the OCD group could potentially support the idea that overactive excitatory signals may be associated with obsessive–compulsive symptoms. Interestingly, we observed cerebellar-visual and striato-limbic communities having the strongest associations with OCI-R scores in the OCD group compared to other communities (*p* < .05, FDR-corrected). This highlights that along with frontal dissociation of striatal and limbic regions, connection between the cerebellum and the visual areas^78^ may be a key feature in OCD. In addition, we also observed regions within the fronto-parieto-default community that included the default mode network (DMN). Although DMN has been thought to be involved in self-awareness and self-referential processing^90^, studies have reported altered resting-state activity of DMN in patients with OCD^15,91^. These findings suggest that observed disconnection and connection of cortical regions may be relevant in OCD.

The pattern of community clustering observed in our study appears to differ from the communities reported by Vaghi and colleagues^22^. While Vaghi et al. (2017) reported a single community that comprises the caudate, putamen, and cerebellar regions in their sample of OCD patients^22^, the similar regions were clustered into separate communities (cerebellar-visual, and striato-limbic communities) in our current sample. These differences may be attributed to the nature of community detection algorithms being non-deterministic, and sample characteristics such as younger age and lower subjective severity of obsessive–compulsive symptoms in our sample. Further research is required to understand the effects of sampling characteristics on the stability of community. Finally, it is also difficult to comprehend the reasons for the non-significant association between inter-individual differences in community size with HAM-A. However, we agree that non-linear and complex associations might exist between them which will benefit from future investigation. Alternatively, it may be possible that anxiety affects networks that are different in OCD.

We observed that OCI-R scores were associated with community size of the fronto-parieto-default and fronto-striato-limbic communities in controls, but the fronto-parieto-default, striato-limbic, and cerebellar-visual in patients with OCD. We no longer observed significant associations in the OCD group when depression and anxiety scores were included in the regression analysis. This implies that for patients with OCD, the communities may not be specific to obsessive–compulsive related symptoms but may also relate to traits of depression and anxiety. Since depression and anxiety often coexist in spectrum of OCD symptoms, the contributions of the identified neural communities towards depression and anxiety require further investigations.

Finally, we would like to highlight the importance of the validation analyses. Accounting for head motion in the rsfMRI data appears to reduce false-positive functional connections that differentiated the two groups, although the key findings remained. The application of GSR in data processing have profound impacts on the results and inferences of the study, mainly due to the fact that GSR introduces artifactual correlations^48,70,71^ that influences group comparisons^72,73^. Our findings were also similar to prior comparison studies involving OCD patients, which reported that the cerebellum and its involvement with other cortical areas in the disorder could only be observed when rsfMRI data are preprocessed without GSR^16,18,80^. Lastly, using an alternate atlas for parcellation did not change our key findings, suggesting that the results observed in the primary analyses were stable.

Even though the current study did not investigate or account for the effects of medication status and comorbidities (18 out of 20 patients were medicated, and all patients did not have comorbid conditions), future investigations could consider the effects of these variables on neural communities. We also acknowledge that the small sample size in the current study may restrict generalizability^92^. Nevertheless, our data demonstrated differences in neural network organization in patients with OCD compared to controls. We showed alterations involving two networks (fronto-striato-limbic and cerebellar-visual) in the OCD group. Further research is required to replicate our findings using larger sample sizes. In particular, it will be interesting to investigate the causal relationship^29,93,94^ between the two communities and demonstrate how the association between areas (cerebellar-visual) potentially compensates the dissociation observed in another network (fronto-striato-limbic).

## Conclusions

In this work, we attempted to investigate the pathophysiology of OCD by maximizing inter-individual differences. We demonstrated that individual-fMRI-subspaces improved detection of functional connectivity differences between OCD and control over conventional-fMRI space. We demonstrated differences in the functional architecture between the two groups using prediction-based approaches. The difference was further investigated with subject-specific community assignments. Communities revealed that frontal dissociation with striatal and limbic regions and cerebellar association with visual regions may be important neural features in OCD. Finally, the association of inter-individual differences in community size with OCI-R score suggests that two more networks (fronto-parietal and default mode) might also serve as biological substrates underpinning the diversity of clinical manifestations in OCD.

## Supplementary Information

Supplementary Information.
