# Supplementary material for: Individual-fMRI-approaches reveal cerebellum and visual communities to be functionally connected in obsessive compulsive disorder
Source: Sci Rep. 2021 Jan 14;11:1354. doi: 10.1038/s41598-020-80346-6 (PMC7809273; doi:10.1038/s41598-020-80346-6)
Supplement: Supplementary file 1 — Supplementary Information. [file 41598_2020_80346_MOESM1_ESM.docx]

**Individual-fMRI-Approaches reveal Cerebellum and Visual Communities to be Functionally Connected in Obsessive Compulsive Disorder**

**Supplemental**

Rajan Kashyap, Goi Khia Eng, Sagarika Bhattacharjee, Bhanu Gupta, Roger Ho, Cyrus SH Ho, Melvyn Zhang, Rathi Mahendran, Kang Sim, SH Annabel Chen

*Study inclusion/exclusion criteria*

Participants were excluded if there is a significant current or past history of alcohol or drug abuse, neurological disease, severe head injury, developmental disorders, learning disorders, visual impairments that cannot be corrected using vision-correcting aids and contraindications for MRI examinations. Patients with OCD fulfilled the Diagnostic and Statistical Manual, 4th Edition (DSM-IV) diagnostic criteria for OCD as assessed using the Structured Clinical Interview for DSM-IV Axis I Disorders (Patient Version) (SCID I-P), and were precluded if they had any history of comorbid conditions. Patients were not excluded for having a history of major depressive disorder unless it preceded the OCD diagnosis. As most OCD patients also have some depressive and anxiety symptoms that do not meet the criteria for clinical depression or other anxiety disorders, the presence of anxiety and depression symptoms were not considered as exclusion criteria if OCD was the primary clinical diagnosis. Controls were assessed using the SCID-I-Non-Patient Version (SCID-I-NP) and were excluded for a history of psychiatric illness or psychiatric disturbances. The details can be obtained from the PhD thesis (<https://dr.ntu.edu.sg/handle/10356/80676>).

*Individual-fMRI-Subspace*

In our prior work, we developed a technique to decompose a subject’s rsfMRI signal into a group-level subspace that is shared across all subjects within the group and an individual subspace that is unique to each subject. A subject’s individual subspace could be isolated by removing the group-level signal from the subject’s rsfMRI signals.

Considering the common group-level signal in both groups appear similar (Figure 1 in main text), instead of extracting the common group-level signal that is specific to each group, one may be tempted to first combine the two groups, then extract the common component to obtain individual fMRI subspace of each subject. We do not recommend this approach. The primary purpose of removing a common and shared group component is to retain features that were specific to the group. By estimating the common group-level in a concatenated group (e.g. OCD + Controls) then removing the “common” component space from the subject’s rsfMRI signal, one runs the risk of removing features that were specific to the other group (OCD or control), and is therefore, conceptually erroneous. Even though the common component in the OCD and control group appear similar (Figure 1 in main text), they were not identical. We explained this using the rsfMRI (116 x 240 matrix) data of a subject from both groups. In Figure S1-A, we plotted the rsfMRI activity across all 116 brain areas of a subject that was randomly selected from the control group (brown curves) and OCD group (blue curves). The rsfMRI activity across the brain areas in the two subjects clearly indicates that both subjects have different resting state signals. In Figure S1-B, we showed the group-level component that was obtained based on the control group (*n* = 22, dotted red line), OCD group (*n* = 20, dashed green line), and data of both groups combined (*n* = 42, blue solid line). Figure S1-B showed that even though the group-level common component in OCD and controls were similar, there were not identical. Furthermore, the group-specific common component deviates from that of the combined group. We performed a two-tailed *t*-test to evaluate differences between the common component of the combined, and specific group (OCD and control), separately. The common component obtained from the combination of the groups was significantly different from each group’s common component (all *p* < .05). Therefore, estimations of the common component should be performed on the specific groups (OCD, Controls) rather than on the concatenated group.


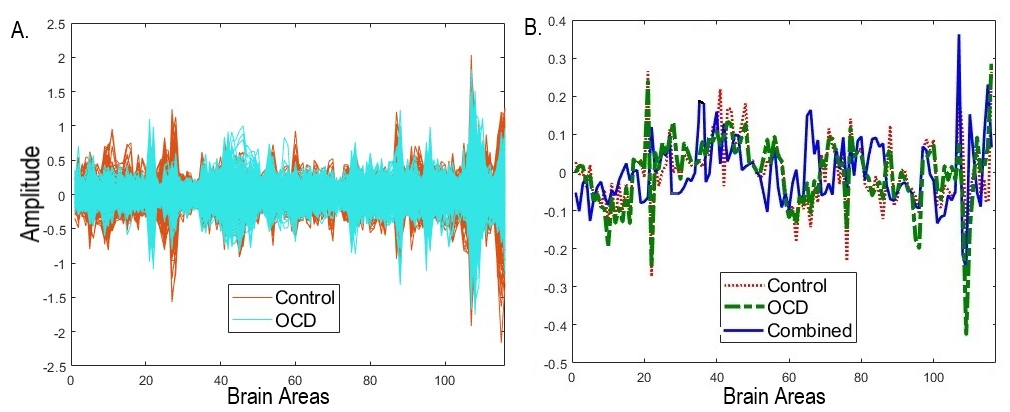


Figure S1. (A) Contribution of the 116 brain areas of a subject selected randomly from the control (brown curves) and OCD (blue curves) group. (B) The group-level common component as obtained from the rsfMRI of all subjects from - control group (*n* = 22), OCD group (*n* = 20), and after combining the control and OCD group (*n* = 42).

*Differences in functional architecture between OCD and controls for other measures*

In the main paper, we evaluated functional connections that were associated with scores derived from the Hamilton Anxiety Rating Scale (HAM-A) and the Obsessive-Compulsive Inventory (Revised; OCI-R); both were significantly different between patients with OCD and controls. We applied elastic-net regression estimation to predict both scores, computed prediction accuracies and the Jaccard Index (JI). JI indicates the amount of common connections between the two groups that were associated with the particular clinical measure.

Here, we applied the same approach to estimate the differences in functional architecture related to depressive scores (assessed using the HDRS). Results were presented in Tables 1S and 2S, respectively. In addition, we also estimated the prediction accuracy values for Y-BOCS (Total). Since Y-BOCS (Total) was only measured for the patients with OCD, we were not able to calculate the prediction accuracy (and Prediction *R^2^*) where inputs from the control group were required. These were mentioned as ‘Not Estimated (NE)’ in Tables 1S and 2S.

Table 1S: Average (± *SD*) of leave-one-out cross-validated prediction accuracy and prediction R^2^ (in brackets and bolded) for the two clinical measures estimated across 1000 permutations.

| Clinical Measure | Prediction accuracy (leave-one-out) | | | |
| --- | --- | --- | --- | --- |
|  | Train-Control-  Test-Control | Train-OCD-Test-OCD | Train-Control-Test-OCD | Train-OCD-  Test-Control |
| HDRS | 0.62 ± 0.27  (**0.38 ± 0.34**) | 0.63 ± 0.24 ( **0.38 ± 0.32**) | 0.08 ± 0.17  (**0.00 ± 0.06**) | 0.06 ± 0.23 (**0.00 ± .06**) |
| Y-BOCS(total) | NE | 0.66 ± 0.24 (**0.43 ± 0.34**) | NE | NE |

Note- NE- Not Estimated

Table 2S: Overlap (Mean ± *SD*) of weighted connections between OCD and control estimated across the permuted samples for the three threshold conditions are shown for HDRS and Y-BOCS (Total).

| Clinical Measure | Top 20% Threshold | | | | Top 50% Threshold | | | | No-Threshold | | | |
| --- | --- | --- | --- | --- | --- | --- | --- | --- | --- | --- | --- | --- |
|  | Control | OCD | OL | JI | Control | OCD | OL | JI | Control | OCD | OL | JI |
| HDRS | 8±16 | 7±22 | 3±2 | 0.08±0.12 | 205±82 | 232±76 | 8±60 | 0.09±0.15 | 980±674 | 1036±496 | 128±96 | 0.14±0.05 |
| Y-BOCS (Total) | NE | 14±17 | NE | NE | NE | 236±92 | NE | NE | NE | 1108 ± 473 | NE | NE |

*Note.* NE- Not Estimated. OL = Overlap.

The number of overlaps is negligible compared to the total number of connections, as also reflected clearly by lower values of Jaccard Index (JI) in Table 2S. Altogether the two tables suggest that the functional architecture may be different in OCD compared to controls.

*Criteria of community selection for each group*

The *mean of the averaged-normalized-entropy* (MANE) for OCD and control was 0.18 and 0.21, respectively. Sixteen community partitions were obtained for OCD and 18 for controls in their respective ranges, i.e., MANE ± 0.02. We observed that when the number of communities in a partition increases above 9, communities with single nodes appear in the subjects. Therefore, the number of communities in each partition ranged from 5 to 9. The values (Mean ± Standard deviation) of the γ for the partitions in OCD and control were 0.01 ± 0.03 and 0.02 ± 0.04, respectively. Similarly, the values of ω for the partitions in OCD and control were 0.29 ± 0.13 and 0.22 ± 0.19, respectively. The values of γ (minimum) for the selected partition in OCD and control was 0.00 and 0.02, respectively. Similarly, the value of ω (maximum) for the selected partition was OCD and control were 0.37 and 0.26, respectively.

*Assigning names to the communities.*

We obtained seven communities for each group. Each community was comprised of a group of areas from the atlas-parcellated (AAL) brain. We assigned a name to the communities based on the areas that clustered together, following naming convention in previous studies. But the names of a few communities may deviate from previous studies (or altered) or may include areas in the neighborhood of previous delineations. This is because (a) limited studies have delineated communities based on AAL-116 parcellation comprising cortical and subcortical areas, and (b) the names are assigned such that the readers can associate the communities with abnormalities of OCD.

The community names for the control group and the regions included within each community were: (1) Fronto-basalganglia – basal ganglia regions (Caudate and Pallidum) and frontal superior medial lobe, (2) Motor-insulo-temporal – pre- and post-central gyrus, insula, heschl gyrus, and temporal pole, (3) Fronto-parietal-default – areas in frontoparietal network and default mode network, (4) Fronto-striato-limbic – regions in the limbic system, striatum (putamen) and inferior frontal lobule, (5) Visual – lingual gyrus, calcarine sulcus, fusiform gyrus, and occipital gyri; (6) Cerebellar-1 – cerebellar lobules 3 to 6, and vermis 1 to 7, and (7) Cerebellar-2 – cerebellar lobules 7 to 10, and vermis 8 to 10.

The community names for the OCD group and the regions included within each community were: (1) Basal-ganglia – basal ganglia regions only (Caudate and Pallidum), (2) Motor-insulo-temporal – similar areas as in controls – pre- and post-central gyrus, insula, heschl gyrus, and temporal pole, (3) Fronto-parietal-default – networks similar in controls – areas in frontoparietal network and default mode network, (4) Striato-limbic – areas of limbic system and putamen, (5) Cerebellar-visual – visual areas, cerebellar lobules 4 to 6 and vermis 6 and 7; (6) Cerebellar-1-Short – cerebellar lobule 3 and vermis 1 to 3 (“short” is added since the areas in this community were fewer than the same Cerebellar-1 community in controls), and finally, (7) Cerebellar-2 – same areas as in controls – cerebellar lobules 7 to 10, and vermis 8 to 10.

Similarities (although not the same) in three communities (Motor-insulo-temporal, fronto-parieto-default, cerebellar-2) were observed between the two groups. Separated communities formed by the putamen and caudate in OCD (compared to intact fronto-striato-limbic, and fronto-basal ganglia communities in controls) potentially highlight the importance of fronto-striato-limbic and fronto-basal-ganglia circuitries in OCD. In general, the observations of frontal areas dissociating from striato-limbic areas, and cerebellar clustering with visual areas as a single community may indicate the importance of these network circuitries in OCD. Importantly, the differences in communities between the OCD and control groups may provide interesting insights regarding the pathophysiology of OCD, although further studies are required to replicate our findings.

*Behavioral association for inter-individual variation in community size*

Inter-individual variation in the number of nodes in a community for the control and OCD group was observed in Figure 2. For any group, the inter-individual variation in the number of nodes in a community was associated with the scores of behavioral measures using linear regression. The model was significant (*p* < .01) across both groups for OCI-R only (Table 3S). The statistical significance of the association of OCI-R with the inter-individual variance in the community size of each community across both group was listed in Table 3S. Two communities (Fronto-parietal-default and fronto-striato-limbic) were significant for controls (*p* < .05, FDR-corrected), whereas three communities (Fronto-parietal-default, striato-limbic and cerebellar-visual) were significant for the OCD sample (p < 0.05, FDR corrected).

Table 3S: Showing the model statistics for the linear regression (behavior ~ inter-individual variation of nodes in Community 1 + inter-individual variation of nodes in Community 2 ....... + inter-individual variation of nodes in Community 7) for the OCI-R behavioral measure

| Model Statistics | Control | OCD |
| --- | --- | --- |
| R-squared | 0.642 | 0.675 |
| Adjusted R-squared | 0.554 | 0.575 |
| F-statistic vs. constant model | 4.73 | 5.32 |
| p-value | 0.0024 | 0.0012 |

It is interesting to note that the fronto-parietal-default community, common in both OCD and control groups, contributes strongly to the association along with control-specific (fronto-striato-limbic) and OCD-specific (Striato-limbic and cerebellar-visual) communities. The findings may indicate the relevance of the fronto-parietal and default mode network community in OCD, as also reported previously.

*Communities with Harvard-Oxford atlas and inter-individual variation in nodes*

Six communities appeared for both OCD and control groups, with the criteria predefined for community selection (Figure 2S). The names assigned to the communities of the control group based on the areas encompassed (16-21) were – (i) Fronto-striatal-limbic + Insulo-temporal, (ii) Default mode, (iii) Primary temporal, (iv) Visual, (v) Cerebellum-1, and (vi) Cerebellum-2. The names assigned to the communities of OCD group were- (i) Fronto + Insulo-temporal, (ii) Default mode, (iii) Primary temporal, (iv) Striato-limbic, (v) Cerebellar-visual, and (vi) Cerebellum-2. Even though the exact communities differ between the Harvard-oxford and AAL atlases, the discrepancy was expected as the areas were specified differently in the atlases. Nevertheless, the disconnection of the striato-limbic areas from frontal areas and a single community that included cerebellar and visual areas were still observed in the OCD group.

The inter-individual variation in the nodes of each community could be observed (inside the inner circle) for both groups. Similar to our previous analysis, these variations were associated with the clinical measures. Models were significant for the OCI-R only. The variation in the default mode community was found significant (*p* < 0.01) for both groups. Interestingly, within the OCD group, inter-individual variation in the cerebellar-visual community was significant (*p* < 0.05, FDR corrected) and not in striato-limbic community (which was found significant with AAL). Similar to our analysis with AAL atlas, the fronto-striato-limbic community showed significant associations with the OCI-R score of control group (*p* < 0.05, FDR corrected).


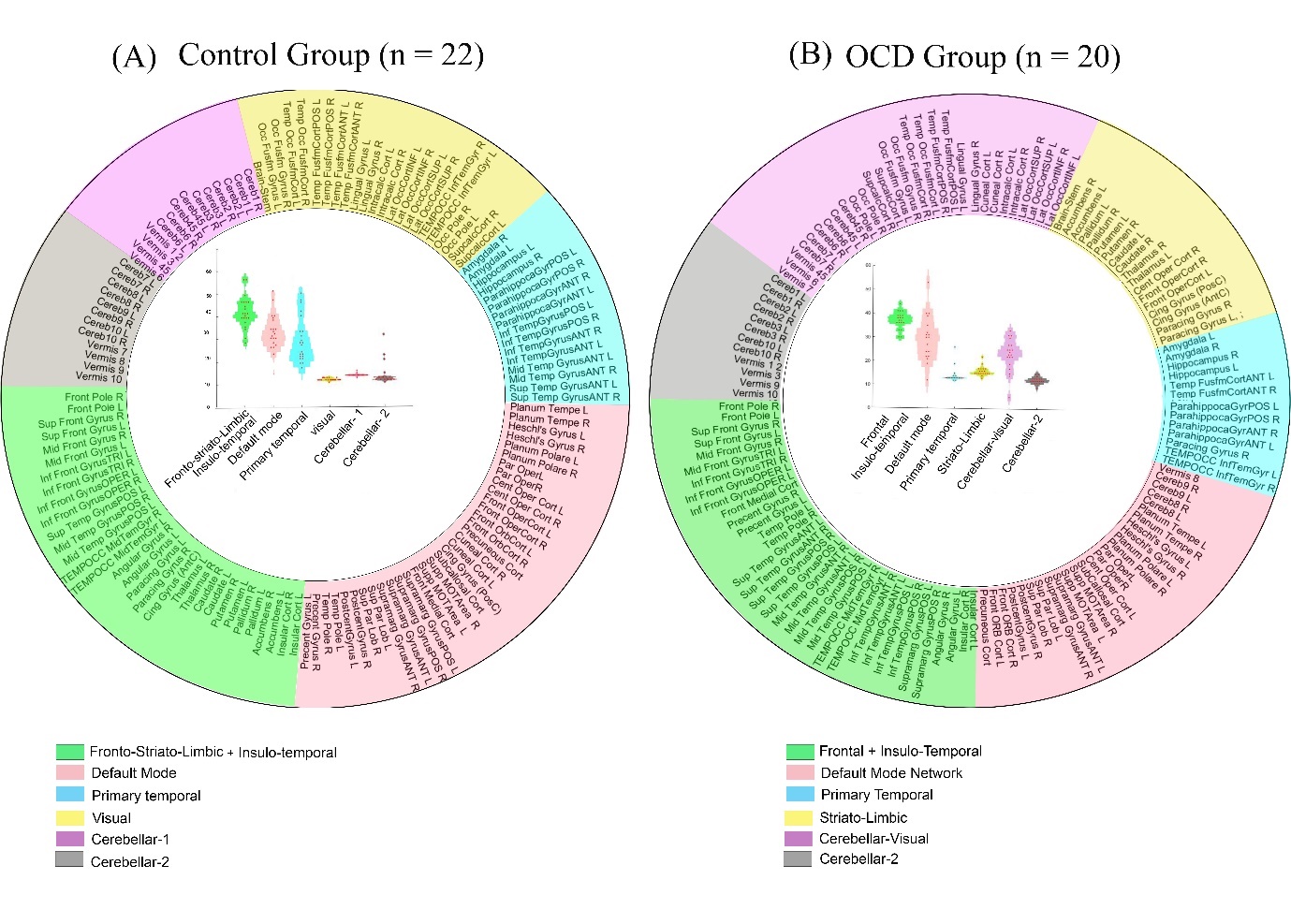


Figure 2S. Six communities from Harvard Oxford atlas are delineated in colors for the (A) control group, and (B) OCD group. Each community (colored region) is named based on the areas encompassed by them. Inside the inner circle, the inter-individual variation (red dots inside the violin plots, where one red dot = one participant) in the number of nodes in each community can be visualized in each group.

*Abbreviation of AAL parcellated brain areas*

The names of a few AAL parcellated brain areas that were abbreviated in the figures are provided in Table 2S. Abbreviated names of the Harvard-Oxford atlas are not provided in this supplement.

Table 2S: Abbreviated names of brain areas and their corresponding full names

| AAL atlas provided the name | Abbreviated name |
| --- | --- |
| Frontal Sup L Frontal Sup R Frontal Sup Orb L Frontal Sup Orb R Frontal Mid L Frontal Mid R Frontal Mid Orb L Frontal Mid Orb R Frontal Inf Oper L Frontal Inf Oper R Frontal Inf Tri L Frontal Inf Tri R Frontal Inf Orb L Frontal Inf Orb R Rolandic Oper L Rolandic Oper R Supp Motor Area L Supp Motor Area R  Frontal Sup Med L Frontal Sup Med R Frontal Med Orb L Frontal Med Orb R  Cingulum Ant L Cingulum Ant R Cingulum Mid L Cingulum Mid R Cingulum Post L Cingulum Post R Hippocampus L Hippocampus R ParaHippocampal L ParaHippocampal R  Occipital Sup L Occipital Sup R Occipital Mid L Occipital Mid R Occipital Inf L Occipital Inf R  SupraMarginal L SupraMarginal R  Paracentral Lob L Paracentral Lob R  Temporal Sup L Temporal Sup R Temporal Pole Sup L Temporal Pole Sup R Temporal Mid L Temporal Mid R Temporal Pol Mid L Temporal Pol Mid R Temporal Inf L Temporal Inf R Cerebelum Crus1 L Cerebelum Crus1 R Cerebelum Crus2 L Cerebelum Crus2 R Cerebelum 3 L Cerebelum 3 R Cerebelum 4 5 L Cerebelum 4 5 R Cerebelum 6 L Cerebelum 6 R Cerebelum 7b L Cerebelum 7b R Cerebelum 8 L Cerebelum 8 R Cerebelum 9 L Cerebelum 9 R Cerebelum 10 L  Cerebelum 10 R | Fr Sup L  Fr Sup R  Fr Sup Orb L  Fr Sup Orb R  Fr Mid L  Fr Mid R  Fr Mid Orb L  Fr Mid Orb R  Fr Inf Op L  Fr Inf Op R  Fr Inf Tri L  Fr Inf Tri R  Fr Inf Orb L  Fr Inf Orb R  Rolan Oper L  Rolan Oper R  Supp Mot A L  Sup Mot A R  Fr Sup Med L  Fr Sup Med R  Fr Med Orb L  Fr Med Orb R  Cing Ant L  Cing Ant R  Cing Mid L  Cing Mid R  Cing Post L  Cing Post R  Hippocam L  Hippocam R  ParaHippo L  ParaHippo R  Occi Sup L  Occi Sup R  Occi Mid L  Occi Mid R  Occi Inf L  Occi Inf R  SupraMargi L  SupraMargi R  Paracent Lob L  Paracent Lob R  Temp Sup L  Temp Sup R  Temp Pol Sup L  Temp Pol Sup R  Temp Mid L  Temp Mid R  Temp Pol Mid L  Temp Pol Mid R  Temp Inf L  Temp Inf R  Cereb Cr1 L  Cereb Cr1 R  Cereb Cr2 L  Cereb Cr2 R  Cereb 3 L  Cereb 3 R  Cereb 4 5 L  Cereb 4 5 R  Cereb 6 L  Cereb 6 R  Cereb 7b L  Cereb 7b R  Cereb 8 L  Cereb 8 R  Cereb 9 L  Cereb 9 R  Cereb 10 L  Cereb 10 R |
